# Metabolic effects of quercetin on inflammatory and autoimmune responses in rheumatoid arthritis are mediated through the inhibition of JAK1/STAT3/HIF-1α signaling

**DOI:** 10.1186/s10020-024-00929-1

**Published:** 2024-10-10

**Authors:** FengQi Zhang, YiYang Zhang, JiaWang Zhou, Ying Cai, ZhiYu Li, Jing Sun, ZhiJun Xie, GuiFeng Hao

**Affiliations:** 1 Key Laboratory of Chinese Medicine Rheumatology of Zhejiang Province, School of Basic Medical Sciences, Zhejiang Chinese Medical University, Binwen Road 548, Binjiang District, Hangzhou, Zhejiang 310053 China; 2 The Second School of Clinical Medicine, Zhejiang Chinese Medical University, Hangzhou, China; 3 The First Affiliated Hospital of Zhejiang Chinese Medical University (Zhejiang Provincial Hospital of Chinese Medicine), Zhejiang Chinese Medical University, Hangzhou, China; 4 Center for General Practice Medicine, Department of Rheumatology and Immunology, Zhejiang Provincial People’s Hospital (Affiliated People’s Hospital), Hangzhou Medical College, Hangzhou, Zhejiang 310014 China

**Keywords:** Quercetin, Rheumatoid arthritis, Inflammatory pathways, Metabolic effects, Molecular docking

## Abstract

**Background:**

Rheumatoid arthritis, a chronic autoimmune disease, is characterized by synovial hyperplasia and cartilage erosion. Here, we investigated the potential mechanism of action of quercetin, the main component of flavonoids, in treating rheumatoid arthritis.

**Object:**

To examine the anti-arthritic effects of quercetin and elucidate the specific mechanisms that differentiate its metabolic effects on autoimmune and inflammatory responses at the synovial cell level.

**Methods:**

We created a collagen-induced arthritis (CIA) model in Wistar rats, which were administered quercetin (50 or 100 mg/kg) continuously for four weeks via stomach perfusion. The arthritis score, histopathological staining, radiological assessment, and serum biochemical parameters were used to study the impact of quercetin on disease improvement. Additionally, immunofluorescence was employed to detect JAK1/STAT3/HIF-1α expression in rat joints. Moreover, the effects of quercetin (20, 40, and 80 µmol/L) on the properties and behavior of synovial fibroblasts were evaluated in an in vitro MH7A cell model using flow cytometry, CCK8, and transwell assays. Further, the mRNA expression levels of inflammatory cytokines IL1β, IL6, IL17, and TNFα were assessed by quantitative real-time PCR. Glucose, lactate, lactate dehydrogenase, pyruvate, pyruvate dehydrogenase, and adenosine triphosphate assay kits were employed to measure the metabolic effects of quercetin on synovial fibroblasts. Finally, immunoblotting was used to examine the impact of quercetin on the JAK1/STAT3/HIF-1α signaling pathway in synovial fibroblasts.

**Results:**

In vivo experiments confirmed the favorable effects of quercetin in CIA rats, including an improved arthritis score and reduced ankle bone destruction, in addition to a decrease in the pro-inflammatory cytokines IL-1β, IL-6, IL-17, and TNF-α in serum. Immunofluorescence verified that quercetin may ameliorate joint injury in rats with CIA by inhibiting JAK1/STAT3/HIF-1α signaling. Various in vitro experiments demonstrated that quercetin effectively inhibits IL-6-induced proliferation of MH7A cells and reduces their migratory and invasive behavior, while inducing apoptosis and reducing the expression of the pro-inflammatory cytokines IL1β, IL6, IL17, and TNFα at the mRNA level. Quercetin caused inhibition of glucose, lactate, lactate dehydrogenase, pyruvate, and adenosine triphosphate and increased pyruvate dehydrogenase expression in MH7A cells. It was further confirmed that quercetin may inhibit energy metabolism and inflammatory factor secretion in MH7A cells through JAK1/STAT3/HIF-1α signaling.

**Conclusions:**

Quercetin’s action on multiple target molecules and pathways makes it a promising treatment for cartilage injury in rheumatoid arthritis. By reducing joint inflammation, improving joint metabolic homeostasis, and decreasing immune system activation energy, quercetin inhibits the JAK1/STAT3/HIF-1α signaling pathway to improve disease status.

## Introduction

The common chronic autoimmune condition, rheumatoid arthritis (RA), has been linked to synovitis and is marked by a persistent inflammatory and hyperplastic state of the synovium, which causes cartilage degeneration and joint bone erosion (Smolen et al. 2016). Research has revealed that the aberrant proliferation of fibroblast-like synoviocytes (FLS) significantly contributes to synovitis and joint destruction (Biniecka et al. 2014). Synovial hyperplasia, neovascularization, and leukocyte extravasation give rise to invasive tumor-like “pannus.” The ensuing vascular system is highly dysregulated, leading to a hypoxic microenvironment in the synovium (Biniecka et al. 2014). This results in abnormal cellular metabolism, mitochondrial dysfunction, and enhanced glycolysis exacerbated by reactive oxygen species production and inflammatory responses. Consequently, lymphocyte proliferation, activation, and migration, as well as imbalances in metabolic homeostasis occur in the joint microenvironment (Fearon et al. 2016). With chronic inflammatory diseases, immune system activation depletes energy, and disruptions in energy metabolism can affect immune system homeostasis. Changes in energy metabolism may be linked to the malignant progression of RA (Jia et al. 2023). Key signaling pathways, such as those mediated by JAK-STAT, Notch, NF-κB, and hypoxia-inducible factor 1α (HIF-1α) are activated by immunoinflammatory cells, leading to synovial invasion (Fearon et al. 2016). RA symptoms are currently managed using disease-modifying anti-rheumatic drugs (DMARDs), non-steroidal anti-inflammatory drugs (NSAIDs), and biological products. Despite their significant therapeutic effects, they display side effects such as drug resistance, vomiting, myelosuppression, infection, and gastrointestinal issues (Yan et al. 2021; Zhao et al. 2021); thus, it is imperative that new safe and highly effective treatments for RA are found.

In RA hypoxic environments, cellular metabolic effects are altered, with oxidative phosphorylation pathways replaced by cellular energy supply from glycolysis (Jia et al. 2023). This results in persistent synovial inflammation and joint damage as vessels proliferate abnormally and the tissue microenvironment changes, leading to upregulated HIF-1α expression (Gaber et al. 2005), which initiates the reprogramming of pyruvate oxidation to the conversion of lactate, which is the end-product of fermentation glycolysis (Luo et al. 2022a, b). Typically, aggressive cancers displaying invasive behavior reside in hypoxic microenvironments and depend on a heightened form of glycolysis to fulfill the greater need for ATP and biosynthetic precursors (Parks et al. 2013). Studies have demonstrated a marked induction of HIF1α expression following the overexpression of STAT3 or the administration of IL6 treatment, while HIF-1α overexpression results in increased levels of JAK1/STAT3(Nagaraju et al. 2013; Boreddy et al. 2011). Therefore, the JAK1/STAT3/HIF-1α pathway is essential for the proliferation of FLS and pannus formation in RA, although further experimental evidence is necessary to ascertain whether quercetin can alter the metabolic effects of the inflammatory pathway in FLS via this pathway.

The traditional Chinese medicine *Herba taxilli* (HT), which consists of the dried leaves and stems of *Taxillus chinensis (DC.) Danser*, is given as a therapy for RA and other inflammatory conditions (Yuan et al. 2021; Shen et al. 2021; Wu et al. 2021). As a major component of HT, the active flavonoid quercetin is considered an indicator of the quality of such extracts and is thought to be responsible for the health benefits produced by HT via its anti-inflammatory, immune regulatory, antioxidative, antiviral, anticancer, and cardioprotective properties (Li et al. 2019; Qin et al. 2022; Guo et al. 2024; Xu et al. 2024). Quercetin acts at several points along the JAK-STAT pathway, inhibiting aberrant activation and displaying significant mediation of inflammation, tumor growth, and cardiovascular disease (Yin et al. 2021). Despite these promising findings, the exact mechanism underlying the effectiveness of quercetin against RA remains elusive.

Here, we aimed to elucidate quercetin’s mechanism of action in treating RA using an array of techniques and experiments both in vitro and in vivo. Additionally, we constructed a CIA rat model to more closely examine quercetin’s anti-inflammatory and joint-protective effects.

## Materials and methods

### Molecular docking

Receptor proteins and ligands were used for molecular docking by selecting the first six core targets and their ligands in the PPI network. Receptor crystal structures were obtained from https://www.rcsb.org/, a PDB database, and water molecules were removed using PyMOL v2.4.1. Subsequently, the optimized receptor structures were hydrogenated to calculate charges using the AutoDock Tools v1.5.7 software, and the data were converted to pdbqt files. The structures of ligands were retrieved in 2D from https://pubchem.ncbi.nlm.nih.gov/ and subsequently converted to 3D and optimized by the ChemBio3D Ultra v17.0 software. The ligand 3D structures were reimported into the AutoDock Tools v1.5.7 software as mol2 files. The AutoDockvina v1.1.2 software was employed for molecular docking, and the PyMOL 2.4.1 software was exploited for the analysis and visualization of data.

### Cell culture

To cultivate MH7A cells, we sourced cryogenic vials from Shanghai Guandao Biological Company, thawed the cells in a water bath at 37 °C while stirring, and disinfected the vials with 75% alcohol before transferring the cells to a fresh sterile tube. After centrifuging at 900 rpm for 5 min, cells were resuspended in DMEM supplemented with 10% FBS and antibiotics (streptomycin, 100 µg/mL; penicillin, 100 U/mL) and then cultured at 37 °C with 5% CO_2_.

### Cell viability assay

To assess the viability of MH7A cells, we utilized the CCK-8 assay in 96-well plates with 5 × 10^3^ cells/well. Cells were stimulated with varying concentrations of quercetin, and a microplate reader was then employed to measure the OD at λ450 nm with a view to constructing a concentration-effect curve and determining the half-maximal inhibitory concentration of quercetin in MH7A cells. In accordance with previous studies, MH7A cells were incubated overnight with IL6 (negative control received medium only) and subsequently subjected to quercetin treatment at 0–100 µM. Supernatants were collected at 24-, 48-, and 72-hours post-treatment with quercetin. Then, 90 µL serum-free medium was added to each well followed by 10 µL CCK-8 solution, and the plates were incubated for a further 1–4 h at 37 °C, after which a microplate reader was used to measure the OD_450_. The inhibition rate of cell growth was calculated as: [OD (no drug) – OD (drug added)]/[OD (no drug) – OD (blank)] × 100%.

### Migration/invasion assays

We used transwell chambers that have porous membranes (6.5 mm, 8-micron pore size; Corning, NY, USA) to evaluate invasion or migration. These chambers were placed in 24-well culture plates. Prior to migration/invasion, cells were starved overnight in serum-free DMEM, after which serum-free DMEM, with or without 250 µg/mL Matrigel^®^ (Corning), was added to cover the chamber surface. Any excess unbound material was aspirated after incubation for 1–2 h at 37 °C. MH7A cells (migration, 2.5 × 104; invasion 5 × 104) were trypsinized for 30 s and then added to the upper chamber in 200 µL 0.4% FBS medium. The lower chamber contained 10% FBS medium, and the same drug treatment was added to both chambers. To allow for migration/invasion through the filter membrane, cells were incubated at 37 °C with 5% CO_2_ for 24 h, fixed for 10 min in 4% paraformaldehyde, and washed with PBS. A cotton swab was used to remove any cells remaining in the upper chamber. A 0.1% crystal violet solution was added for 10 min to stain the migrated/invaded cells on the bottom surface of the filter membrane before washing off the background dye. Quantitation of stained cells was performed using a light microscope (magnification ×20). The average cell number per nine random fields was determined for each assay.

### Apoptosis determination

To assess the influence of quercetin on MH7A cell apoptosis, we employed the FITC Annexin V Apoptosis Detection kit (BD, 556547). Cells were treated under different conditions, incubated in DMEM supplemented with 10% FBS, trypsinized, and then washed twice with PBS. Next, cells were resuspended at 1 × 10^6^ cells/mL in a 100-µL volume of binding buffer, to which 5 µL each annexin V-FITC and PI were pipetted. Following a 15-minute incubation at room temperature, 400 µL binding buffer was added to the cells. Flow cytometry (Beckman Coulter, CytoFlex S) was used to measure the number of apoptotic cells.

### Determination of glucose, LA, LDH, PDH, PA, and ATP levels

The glucose (Glucose; Solarbio, BC2505), lactate (L-LA; Solarbio, BC2235), pyruvate (PA; Solarbio, BC2200), lactate dehydrogenase (LDH; Solarbio, BC0685), pyruvate dehydrogenase (PDH; Solarbio, BC0385), and adenosine triphosphate (ATP; Solarbio, BC0305) levels in MH7A cells were measured using the respective assay kits. MH7A cells treated for 24 h with varying drug concentrations were collected in 1.5-mL centrifuge tubes prior to the experiment.

### RT-qPCR

RNAiso Plus reagent was employed for total RNA extraction from MH7A cells. Subsequently, cDNA synthesis was performed with the use of the iScript cDNA synthesis kit (Bio-Rad, 1708891), and relative quantitation of target genes was carried out using the SYBR™ Green premix pro-Taq qPCR Kit (Accurate Biology, AG11701), with GAPDH serving as an internal reference. PCR was carried out using the LightCycler 96 (Roche, Basel, Switzerland). Listed below are the sequences of the primers utilized: HIF-1α, For 5’-CCATTAGAAAGCAGTTCCGCAAGC-3’ and Rev 5’-GTGGTAGTGGTGGCATTAGCAGTAG-3’; IL1β, For 5’- CCAGGGACAGGATATGGAGCA-3’ and Rev 5’-TTCAACACGCAGGACAGGTACAG-3’; IL6, For 5’-AAGCCAGAGCTGTGCAGATGAGTA-3’ and Rev 5’-TGTCCTGCAGCCACTGGTTC-3’; IL17, For 5’-GACTCAGGCTTCCTTTGG-3’ and Rev 5’-GCTCCTTTCTGGGTTGTG-3’; GAPDH, For 5 ‘-GAACGGGAAGCTGG-3’ and Rev 5 ‘-GCCAGCTTCACCTCCTTCT-3’.

### Immunoblotting

MH7A cells were subjected to lysis on ice in RIPA buffer supplemented with phosphatase and protease inhibitors to extract total protein. Following centrifugation at 4 °C, 12,000 rpm for 10 min, total protein concentration was measured using the Pierce™ BCA kit. An equal protein concentration from each sample was then boiled for 10 min in 5× loading buffer and separated by SDS-PAGE (Epizyme). Proteins were transferred to PVDF membrane, which was subjected to blocking in 5% skimmed milk/PBS, and then incubated with primary antibodies overnight at 4 °C. The following day, after washing three times, secondary antibodies were applied to the membrane for 1 h. A ChemiDoc imaging system (Bio-Rad) was used to visualize protein bands. The primary antibodies used were raised against JAK1, STAT3, HIF-1α, and GAPDH. GAPDH served as the loading control for normalization.

### Creation of the collagen-induced arthritis (CIA) rat model and drug therapy

Six-week-old Wistar rats (male; 160 ± 20 g) were sourced from the Laboratory Animal Service Center at the Zhejiang University of Traditional Chinese Medicine in Hangzhou, China. Rats were housed at 22 ± 2 °C and 55 ± 2% humidity under a 12 h/12 h light/dark cycle and had free access to drinking water and standard chow. The animal experiments were conducted after gaining approval from the Animal Ethics Committee at Zhejiang Chinese Medical University (IACUC-20221008-06). The rats were adaptively fed for one week prior to establishing the collagen-induced arthritis (CIA) model using literature methods (Rosloniec et al. 2010). The brief description is as follows: six rats were selected as control group, and the others were primarily immunized with CII emulsified in FCA. About 200 µL of bovine CII emulsion (2.0 mg/ml) was injected intradermally at the base of the tail. Fourteen days post-immunization, CIA rats possessing an arthritis score greater than 6 were assigned randomly to three groups: model (CIA, *n* = 6), model + 50 mg/kg quercetin (*n* = 6), and model + 100 mg/kg quercetin (*n* = 6). These concentrations were selected according to literature recommendations, which are equivalent to doses of 0.5 and 1 mg/k/day for normal subjects (El-Said et al. 2022). Quercetin (> 98.0% purity), a major bioactive constituent of HT, was bought from Shanghai Aladdin Biochemical Technology Co., Ltd. (Sigma, USA, CAS No.: 117-39-5). Quercetin powder was resuspended in distilled water to create a suspension for injection. Control and model rats were given saline in an equal volume.

### Assessment of arthritis severity

After the initial immunization, we evaluated the frequency and intensity of arthritis every three days, while also recording changes in body weight at the same intervals. We utilized a modified scoring system to grade arthritis severity in each limb from 0 to 4, with 0 indicating no swelling and 4 indicating swelling throughout the whole foot, including the ankle area. Scores of 1, 2, and 3 indicated swelling of the lesser toe joint, toe joint and sole, and foot below the ankle, respectively. The scores for all four extremities were summed to calculate the arthritis score for each rat, with a total score of 4 or higher indicating successful construction of the CIA model. The maximum possible score was 16 (4 × 4).

### Histopathological examination and radiological assessment

Upon conclusion of the animal experiment, synovium was extracted from the joints of both the ankle and knee and subjected to Micro-CT (BrukermicroCT Kontich, Belgium) imaging with a view to observing any morphological changes present. Furthermore, the ankle joints and synovium were flushed with PBS and fixed using a 4% paraformaldehyde solution. Decalcification of the ankle joints was achieved using an EDTA solution. Subsequently, samples were paraffin embedded, sliced into sections, and subjected to hematoxylin and eosin (H&E) staining to examine changes present in the ankle and synovium.

### Enzyme-linked immunosorbent assay and blood biochemical test indicators

TNF-α (CSB-E11987r), IL-1β (CSB-E08055r), IL-6 (CSB-E04640r), and IL-17 (CSB-E07451r) levels in serum samples taken from rats were determined using an ELISA kit (Huamei, China). A microplate reader was employed to assess absorbance values (TECAN, INFINITE 200 PRO, Austria). In addition, the levels of ALT, AST, ALB, ALP, CREA, and BUN were measured on an automated biochemical analyzer.

### Statistical analysis

All data are expressed as the mean ± SD. CytExpert and GraphPad Prism 9.0 were exploited for statistical analysis. Inter-group differences were evaluated by one-way ANOVA and Dunnett’s *post-hoc* test, while intra-group comparisons were made using a Student’s *t*-test. Statistical significance was considered as *P* < 0.05, *P* < 0.01, or *P* < 0.001. Repeats were biological replicates.

## Results

### Quercetin inhibits MH7A cell proliferation

**Fig. 1:**
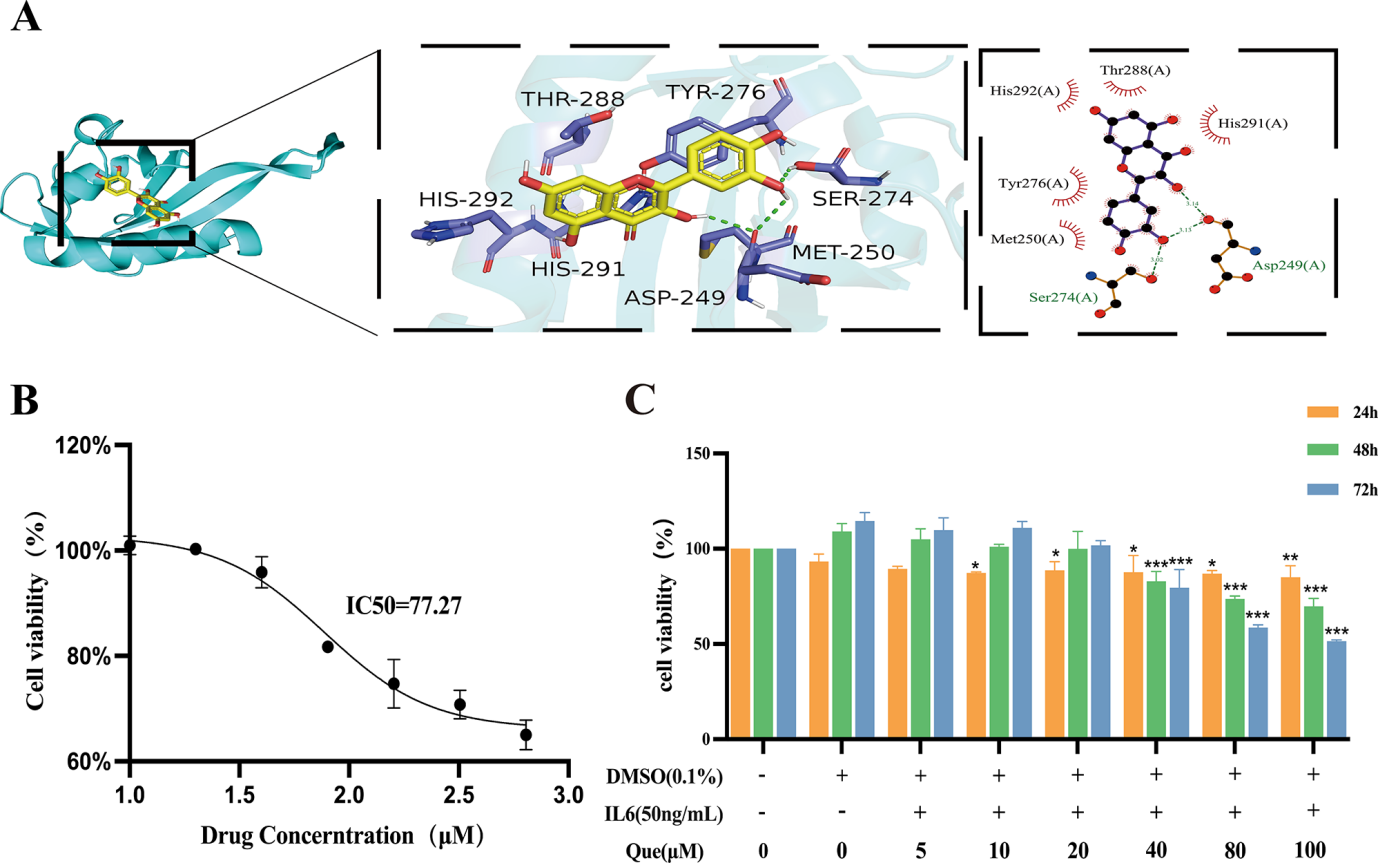
Quercetin impedes the proliferation of MH7A cells. (**A**) Molecular docking of quercetin at HIF-1α. (**B**) Antiproliferative effect of 5, 10, 20, 40, 80, and 100 µM quercetin on MH7A cells following the addition of 50 ng/mL IL6 (*n* = 3) (**C**) IC_50_ of quercetin. Data are expressed as the mean ± SD. Results indicate a significant improvement in comparison with the arthritis group (*P < 0.05, **P < 0.01, ***P < 0.001)

Synovial fibroblasts generate a hypoxic microenvironment in inflammatory conditions, wherein HIF-1α is crucial in regulating the balance between oxygen supply and demand (Liu et al. 2024). HIF-1α facilitates glycolysis and is critical in the metabolic reprogramming of synovial fibroblasts, and it exhibits a high affinity for quercetin in molecular docking, as shown in Fig. 1A. To test the predicted molecular mechanism of quercetin identified through in vivo experiments, CCK-8 and inhibitory assays were conducted. The results indicate that quercetin dose-dependently hinders MH7A cell proliferation. Cells were exposed to varying concentrations of quercetin (5, 10, 20, 40, 80, and 100 µmol/L) in serum-free medium for 24, 48, and 72 h. Treatment with 5 and 10 µmol/L quercetin did not have a significant impact on cell viability, but concentrations equal to or greater than 77.27 µM significantly slowed the proliferation of MH7A cells (*P* < 0.05). These preliminary findings led us to select three concentrations of quercetin (20, 40, and 80 µmol/L) for subsequent experiments over 24 h.

### Quercetin impedes the migration and invasion of MH7A cells and induces apoptosis to reduce inflammatory factor secretion

**Fig. 2:**
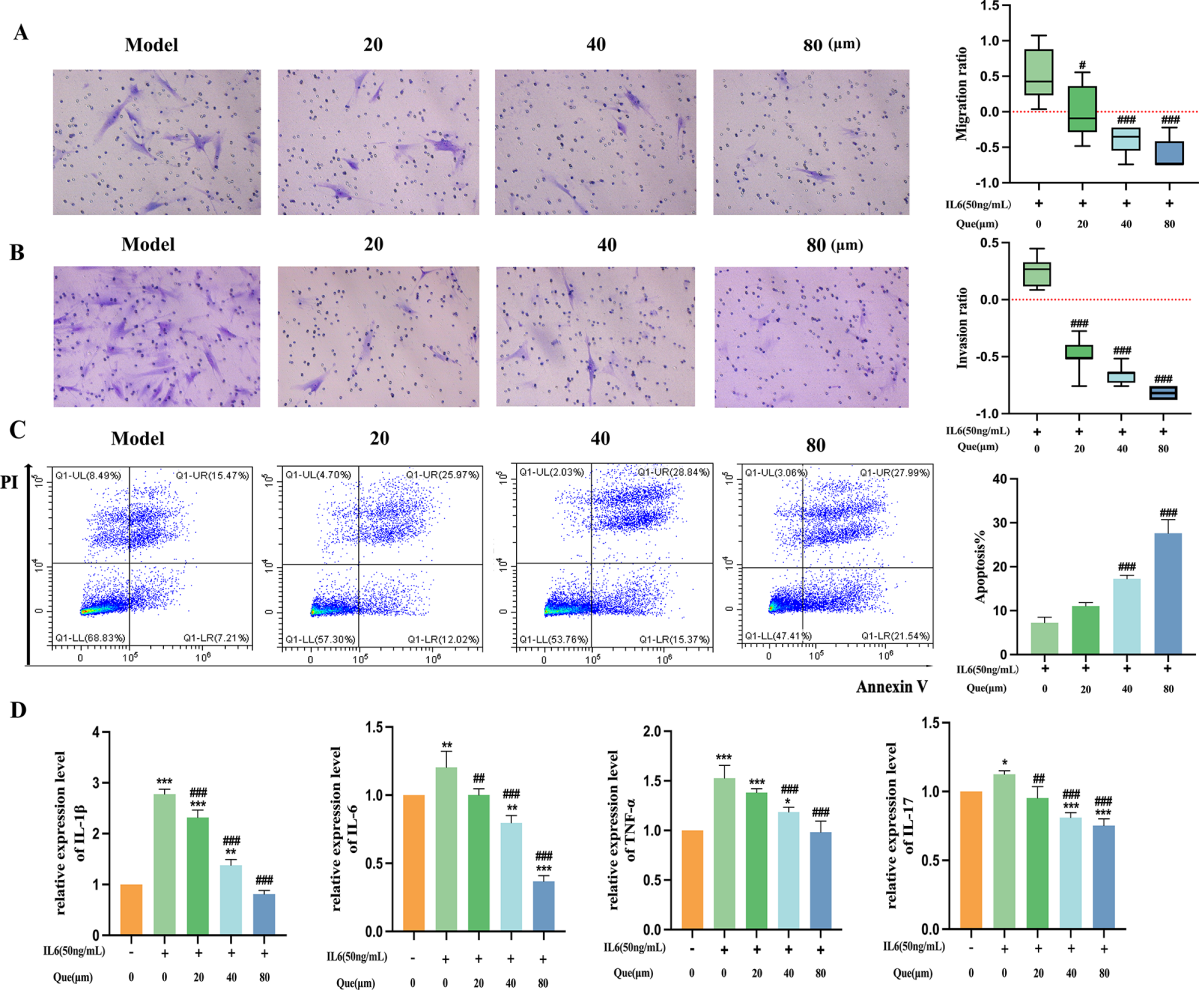
Quercetin impedes migration and invasion and induces apoptosis in MH7A cells. (**A**, **B**) Assessment of MH7A cell migration and invasion using a transwell assay following quercetin treatment (*n* = 5). (**C**) MH7A cells in active apoptosis were detected by flow cytometry following annexin V-FITC/PI staining (*n* = 3). (**D**) IL-1β, IL-6, IL-17, and TNF-α mRNA expression as assessed by RT-qPCR (*n* = 3). Data are expressed as the mean ± SD. Results indicate a significant improvement in comparison with the arthritis (^#^*P* < 0.05, ^##^*P* < 0.01, and ^###^*P* < 0.001) or control (*P < 0.05, **P < 0.01, ***P < 0.001) groups

Synovial cell migration and invasion may lead to bone and joint damage in patients with RA. In order to evaluate the effect of quercetin on MH7A cell migration, invasion, and apoptosis, transwell techniques and flow cytometry were employed. Figure 2AB illustrates that quercetin (20, 40, and 80 µmol/L) dose-dependently suppressed MH7A cell migration and invasion.

In Fig. 2C, late apoptotic, necrotic, viable, and early apoptotic cells are represented by quadrants Q1, Q2, Q3, and Q4, respectively, which suggests that apoptosis may hinder cell migration and invasion. It is clear from the data that quercetin induced apoptosis in MH7A cells, and the degree of apoptosis increased with higher concentrations of quercetin. Additionally, RT-qPCR data in Fig. 2D demonstrates that quercetin reduced the expression of TNF-α, IL1β, IL6, and IL17 at the mRNA level in comparison with the arthritis group (*P* < 0.05, *P* < 0.001).

### Quercetin decreases IL6-induced energy metabolism and oxidative stress in MH7A cells

**Fig. 3:**
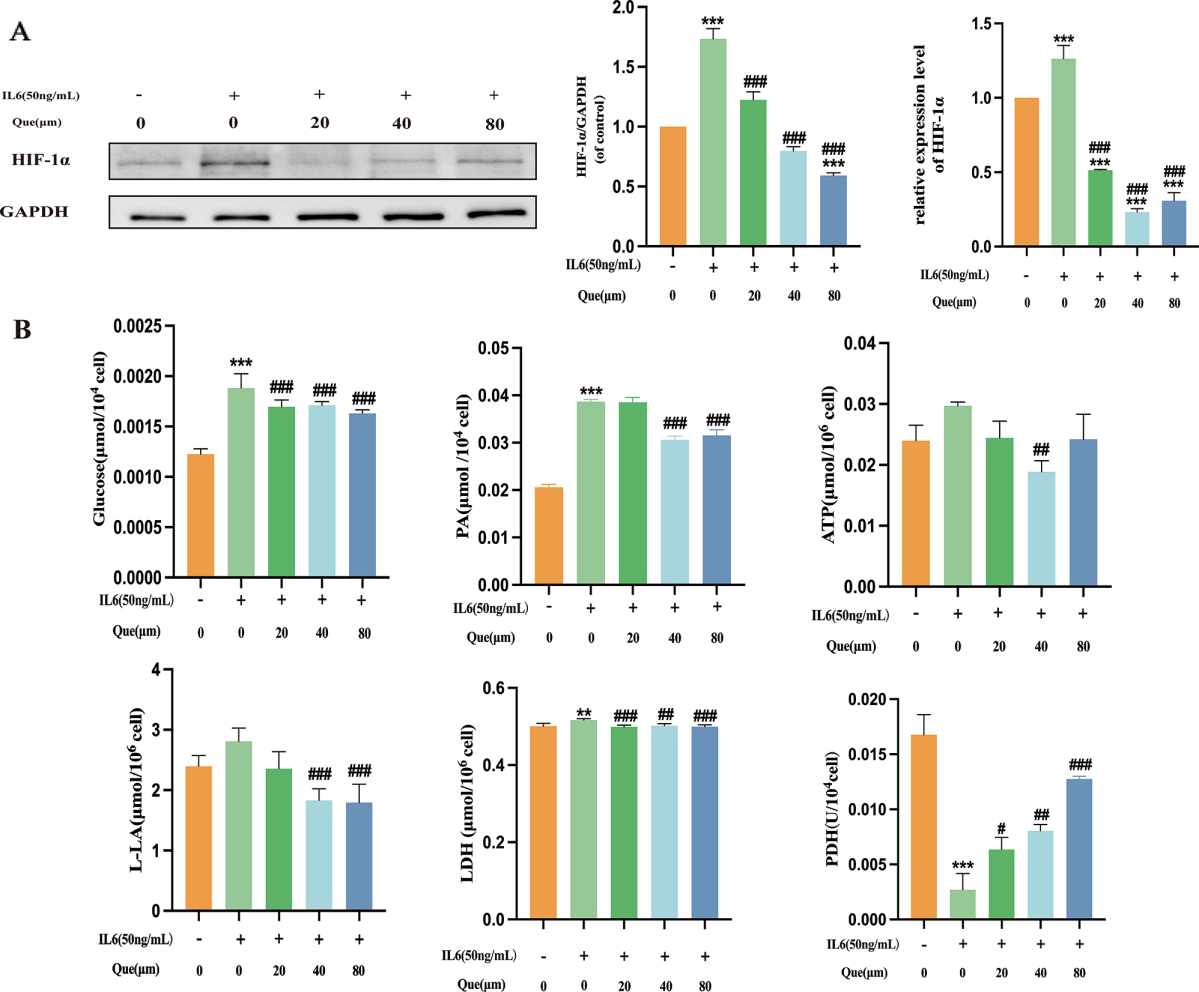
Quercetin decreases IL6-induced oxidative stress and energy metabolism in MH7A cells. (**A**) Quercetin decreases the expression levels of HIF-1α in MH7A cells at both the protein and mRNA levels (*n* = 3). (**B**) Effect of quercetin on glucose, LDH, LA, PA, PDH and ATP levels in MH7A cells (*n* = 4). Data are expressed as the mean ± SD. Results indicate a significant improvement in comparison with the arthritis (^#^*P* < 0.05, ^##^*P* < 0.01, and ^###^*P* < 0.001) or control (*P < 0.05, **P < 0.01, ***P < 0.001) groups

To assess the significance of HIF-1α function in RA, we evaluated its protein and mRNA expression levels by immunoblotting and RT-qPCR, respectively. Figure 3A shows that HIF-1α protein and mRNA expression levels were markedly increased in the model group as compared with the normal group, highlighting an important role for HIF-1α in RA. We noticed that the expression level of both HIF-1α mRNA and protein was significantly reduced by quercetin, emphasizing the involvement of HIF-1α in RA.

We then assessed the impact of HIF-1α on glycolysis in MH7A cells to investigate whether quercetin regulates energy metabolism via HIF-1α. Using specific detection kits, we assessed the levels of Glucose, LDH, LA, PA, PDH, and ATP in MH7A cells. Our findings demonstrate that the application of quercetin markedly decreased the levels of glucose as shown in Fig. 3B, LDH, LA, PA, and ATP, while increasing PDH levels (*P* < 0.05, *P* < 0.01, *P* < 0.001). ATP production efficiency, a crucial glycolysis index, was markedly lower in the quercetin group than in the model group. Furthermore, quercetin administration resulted in a dose-dependent reduction in lactate production, a significant product of glycolysis. Quercetin also suppressed LDHA and increased PDH, which serves as a crucial mechanism to catalyze the most important step of glycolysis.

### Quercetin inhibits IL6-triggered JAK1/STAT3/HIF-1α signaling

**Fig. 4:**
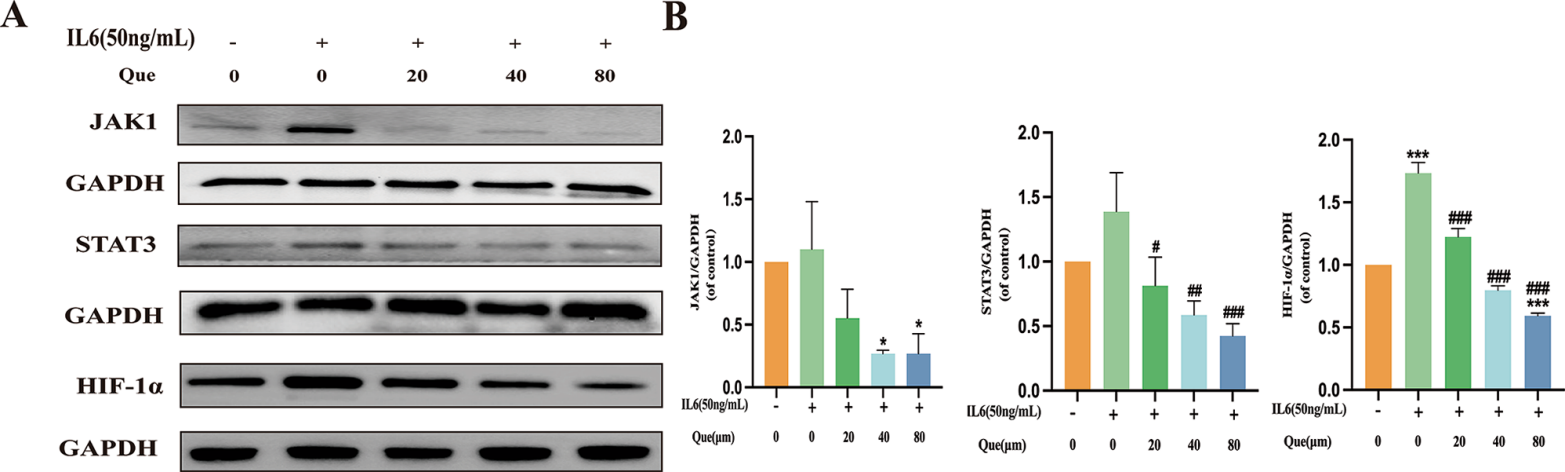
Effect of quercetin on JAK1/STAT3/HIF-1α signaling in MH7A cells. (**A**) Crosstalk in the JAK1/STAT3/HIF-1α signaling pathway. Protein expression levels as assessed using immunoblotting (*n* = 3). (**B**) JAK1, STAT3, and HIF-1α protein expression levels were assessed using immunoblotting (*n* = 3). Data are expressed as the mean ± SD. Results indicate a significant improvement in comparison with the arthritis (*P < 0.05, **P < 0.01, ***P < 0.001) or control (^#^*P* < 0.05, ^##^*P* < 0.01, and ^###^*P* < 0.001) group

Our investigation delved deeper into the regulation of upstream mechanisms in MH7A cells by quercetin. We know that flavone compounds can have an impact on synovial energy metabolism and proinflammatory mediators in RA through JAK-STAT pathway blockade. With this knowledge in mind, we hypothesized that quercetin impacts HIF-1 A via the JAK/STAT pathway to improve inflammatory infiltration of synoviocytes and how energy imbalance. We used an in vitro model to demonstrate that quercetin significantly suppressed JAK1/STAT3/HIF-1α protein expression levels (*P* < 0.05, *P* < 0.01, *P* < 0.001). These data suggest that quercetin can suppress inflammatory responses and joint destruction by modulating JAK1/STAT3/HIF-1α signaling (Fig. 4).

### Quercetin improves the inflammatory response and bone destruction in the ankle joints of rats with collagen-induced arthritis (CIA)

**Fig. 5:**
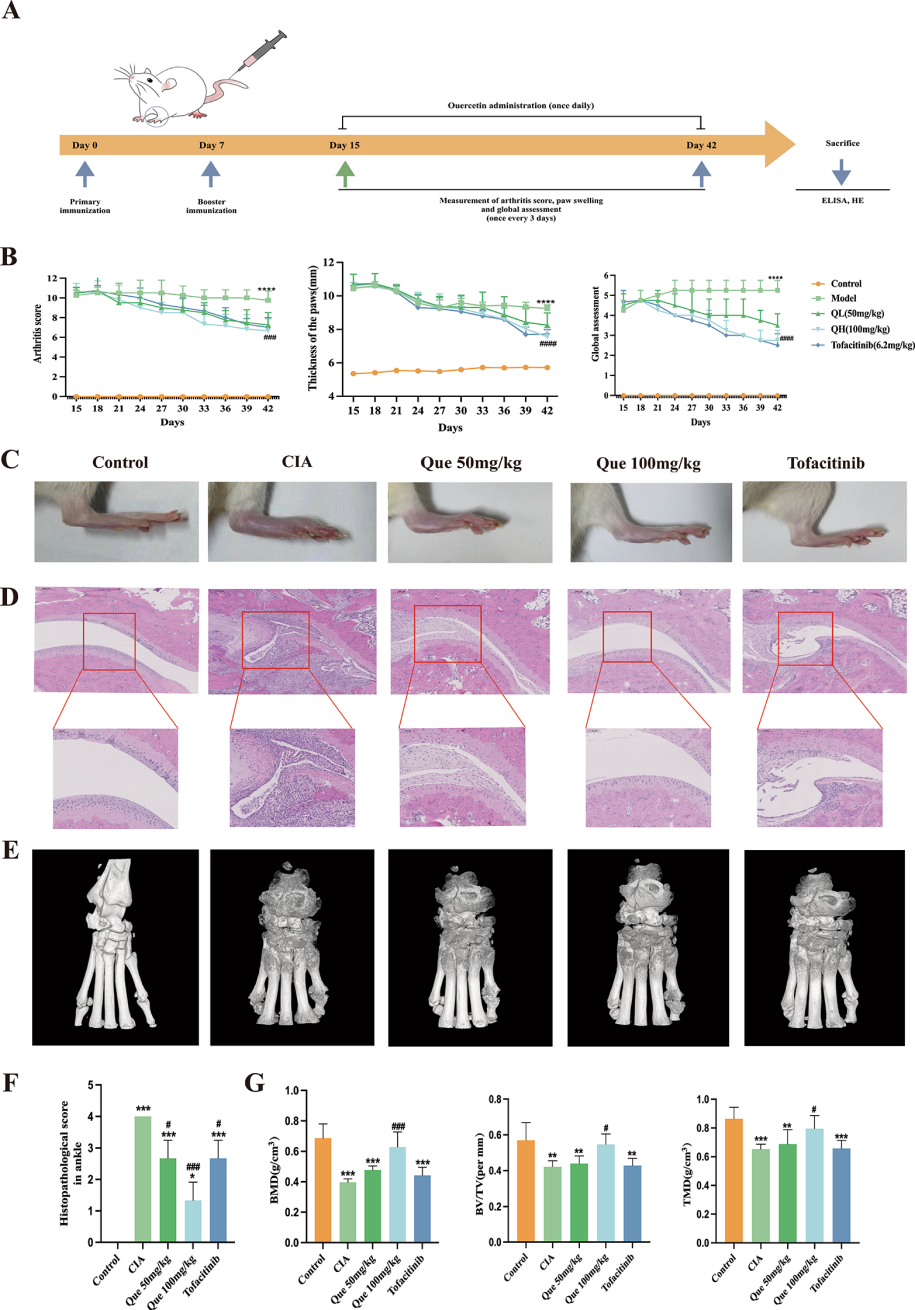
Quercetin improves the destruction of bone in CIA rats. (**A**) The schedule for establishing the CIA model and performing the drug treatment regimen (Que and Tofacitinib) is depicted as a schematic diagram. (**B**) Comparison of the changes in the scores indicating arthritis, paw swelling, and global assessment among the groups of rats (*n* = 7). (**C**) Representative appearance of each ankle. (**D**) Comparison of the histopathology of rodent ankle joints among the groups (*n* = 7). (**E**) Comparison of the micro-CT images of rodent ankle joints among the groups (*n* = 7). (**F**) Histopof the ankle joints among the groups of rats (*n* = 3). (**G**) Morphometric data of TMD (g/mm^3^), BMD (mm^2^) and BV/TV (%) in knee and ankle joints (*n* = 6). Data are expressed as the mean ± SD. Results indicate a significant improvement in comparison with the arthritis (*P < 0.05, **P < 0.01, ***P < 0.001) or control (^#^*P* < 0.05, ^##^*P* < 0.01, and ^###^*P* < 0.001) group

The effectiveness of quercetin in treating CIA in rats was evaluated (Fig. 5A). Throughout the experiment, the negative control group exhibited significant swelling, erythema, and ankylosis of the hind paws, as evaluated by arthritis scores (Fig. 5B, left) and paw swelling (Fig. 5B, middle), as compared with normal rats. The treatment of positive control rats with Tofacitinib resulted in a reduction of RA symptoms, as indicated by a decrease in arthritis score (*P* < 0.01) and paw volume (*P* < 0.01) as compared with model rats. Interestingly, both the Que50 mg/kg and Que100 mg/kg groups demonstrated a marked reduction in arthritis score (*P* < 0.01) and paw volume (*P* < 0.01) after day 27, which was comparable to the response seen with Tofacitinib.

The macroscopic changes in the paws of CIA rats were examined following different treatments (Fig. 5C), after which the pathology present in the ankle joints was assessed by H&E staining of tissue sections. Significant RA symptoms, such as swelling and redness, were seen in the rodent paws in the model group; however, following treatment with quercetin or Tofacitinib, RA symptoms were alleviated to varying degrees. Additionally, H&E staining revealed substantial changes in pathology, which included inflammatory responses, hyperplastic synovium, and destruction of cartilage, in the rodent ankle joints in the control group (Fig. 5B). Notably, both quercetin and Tofacitinib administration resulted in significant improvements in these pathological changes.

Moreover, we conducted ankle-joint micro-CT scans on rats in the normal, negative control, positive control, and treatment groups. The outcomes presented in Fig. 5E demonstrate that the joint configuration of healthy rats was distinct, with a moderate joint gap and even articular surface. Conversely, the rodent ankle joints in the negative control group displayed an uneven bone surface, reduced joint gap, severe joint erosion-like alterations, and fusion of the articular surface. Remarkably, Que100 mg/kg administration resulted in a substantial reduction in ankle joint destruction, similar to that achieved with Tofacitinib treatment.

### Quercetin reduces the serum levels of IL1β, IL6, IL17, and TNF-α to alleviate bone destruction in CIA rats

**Fig. 6:**
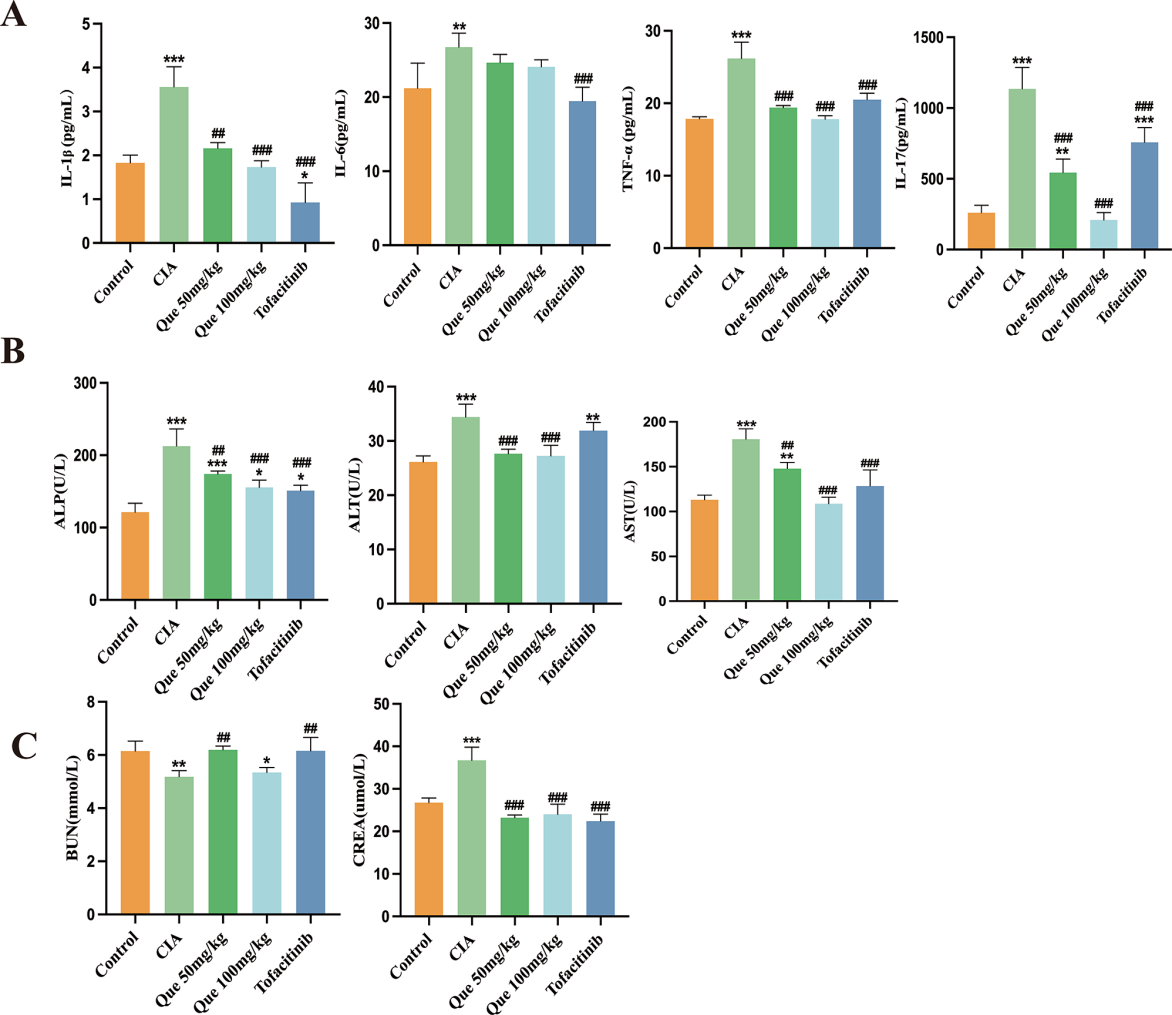
Quercetin decreases inflammatory factor expression in CIA rats. (**A**) Pro-inflammatory cytokine levels, including IL-1β, IL-6, IL-17, and TNF-α (*n* = 4), were assessed using ELISA. (**B**) Effects of quercetin on liver function parameters in CIA rats. (**C**) Quercetin’s effects on renal function parameters. Data are expressed as the mean ± SD. Results indicate a marked improvement in comparison with the arthritis (*P < 0.05, **P < 0.01, ***P < 0.001) or control (^#^*P* < 0.05, ^##^*P* < 0.01, and ^###^*P* < 0.001) group

The progression of RA is notably affected by several pro-inflammatory cytokines, with IL-1β, IL-6, IL-17, and TNF-α being particularly important. Immune cells and synoviocytes are activated by IL-6 and IL-1β, the secretion of which is further promoted by TNF-α, which facilitates RA development (Kugler et al. 2023). Moreover, osteoclast differentiation and proliferation are accelerated by IL-17, leading to bone resorption and eventual destruction of articular cartilage and bone. To determine whether quercetin influences pro-inflammatory cytokine secretion, ELISA was used to measure their levels. As depicted in Fig. 6A, IL-1β, IL-6, IL-17, and TNF-α levels were markedly increased in the CIA group (*P* < 0.05, *P* < 0.01, or *P* < 0.001), while their release was inhibited dose-dependently by quercetin administration (*P* < 0.05, *P* < 0.01, or *P* < 0.001). Notably, the release of inflammatory cytokines was also reduced following Tofacitinib administration, but quercetin offered better protection of the kidneys and liver (as shown in Fig. 6B, C). Overall, these results indicate that quercetin can alleviate symptoms in CIA rats by reducing hind paw swelling and improving joint inflammation and destruction (Fig. 6).

### Quercetin reduces JAK1/STAT3/HIF-1α expression in the ankle joints of CIA rats

**Fig. 7:**
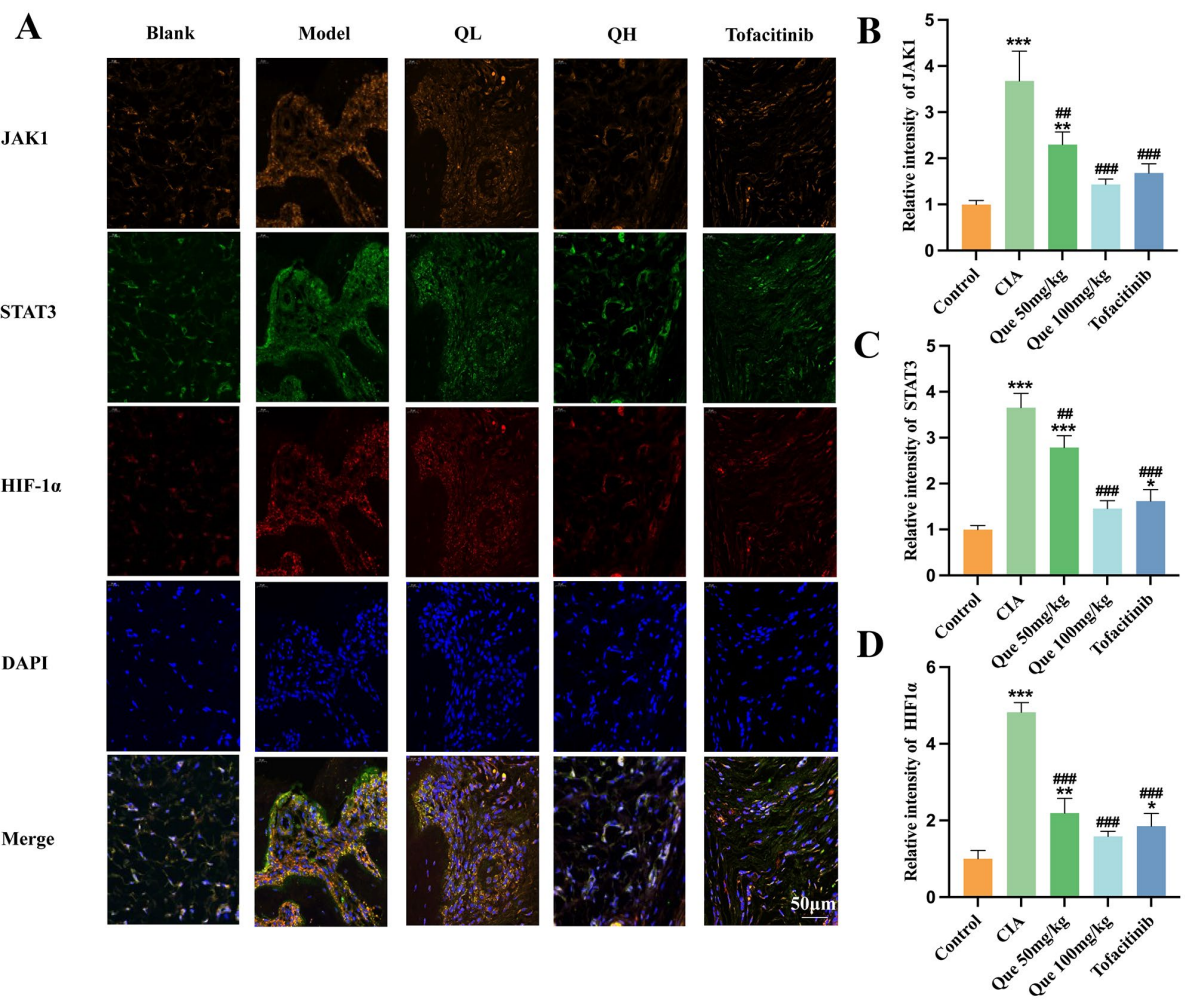
Immunofluorescence of JAK1/STAT3/HIF-1α in the ankle joints of CIA rats. (**A**) Effects Levels of Immunofluorescence in the ankle joints of CIA rats, including JAK1, STAT3, and HIF1α. (**B**-**D**) Levels of Immunofluorescence in the ankle joints of CIA rats, including JAK1, STAT3, and HIF1α (*n* = 3). Data are expressed as the mean ± SD. Results indicate a marked improvement in comparison with the arthritis (*P < 0.05, **P < 0.01, ***P < 0.001) or control (^#^*P* < 0.05, ^##^*P* < 0.01, and ^###^*P* < 0.001) group

Our study conducted a more comprehensive investigation into quercetin’s local effect within the ankle joints of CIA rats. It has been documented that JAK/STAT pathways are critically involved in cell–cell interactions during RA pathogenesis, which results in inflammation and proliferation of the synovium, secretion of autoantibodies, and joint destruction. Quercetin can affect pro-inflammatory mediators in RA synovium through JAK/STAT pathways, and HIF-1α activation can lead to the upregulation of proinflammatory mediators in RA. Thus, we evaluated JAK1/STAT3/HIF-1α expression in rodent ankle joints in the CIA group using immunofluorescence and discovered a marked increase as compared with the normal group (Fig. 7), indicating a state of joint erosion. However, the low- and high-dose quercetin groups showed dose-dependent relief of joint bone destruction in CIA rats. These findings suggest that quercetin may suppress inflammatory responses and joint destruction by modulating JAK1/STAT3/HIF-1α signaling.

## Discussion

The synovial intimal layer relies on fibroblast-like synoviocytes to preserve the joints’ integrity and functionality. These synoviocytes are critical components and are essential for maintaining joint health (Nygaard et al. 2020). In the case of RA, activation of these cells results in the accumulation of inflammatory factors that disrupt local energy metabolism. Additionally, the immune system requires more energy than the body’s resting state; therefore, RA patients often experience cellular metabolic dysfunction due to reduced oxygen concentration (Binymin et al. 2011). The dysregulation of the synovial microvascular system in RA results in inefficient oxygen perfusion, creating a severely hypoxic microenvironment that exacerbates the high metabolic demand of stromal and immune cells (Fearon et al. 2022). Thus, balancing inflammation and the immune system and correcting energy metabolic disorders is crucial in the treatment and management of RA.

Quercetin is a natural product found in plants (Takahashi et al. 2019), and studies have demonstrated its effective suppression of inflammation in various diseases, including malignancy (Murakami et al. 2008), diabetes (Yan et al. 2023), and atherosclerosis (Luo et al. 2022a, b). The presence of synovitis and hyperproliferation of synoviocytes, along with the infiltration of immune cells and destruction of cartilage, led to joint damage and stiffness in CIA rats. These features were found to be closely related to clinical RA. Previous work has shown that quercetin significantly reduces TNF-α production in CIA rats (Gardi et al. 2015) and protects mice from arthritis (Haleagrahara et al. 2017). Moreover, quercetin/casein microparticles improve oxidative stress in arthritic rats via an anti-inflammatory-independent action (Souza et al. 2021). Here, we employed a CIA rat model to examine the efficacy of quercetin and found an improved total body score, arthritis score, and degree of foot swelling in CIA rats, suggesting that quercetin may enhance quality of life. Additionally, quercetin decreased the serum levels of the inflammatory cytokines, IL-1β, IL-6, IL-17 and TNF-α. Moreover, our experiments demonstrate that quercetin is safe and protective for the kidneys and liver in vivo. We conducted ankle-joint micro-CT scans on rats in the normal, negative control, positive control, and treatment groups. The outcomes presented in Fig. 5E demonstrate that the joint configuration of healthy rats was distinct, with a moderate joint gap and even articular surface. Conversely, the rodent ankle joints in the negative control group displayed an uneven bone surface, reduced joint gap, severe joint erosion-like alterations, and fusion of the articular surface. Remarkably, Que100 mg/kg administration resulted in a substantial reduction in ankle joint destruction comparable to that achieved with Tofacitinib treatment; thus, our findings suggest that quercetin has significant potential for treating RA.

The migration of synovial fibroblasts to unaffected joints is the primary cause of cartilage destruction in patients with RA (Smith et al. 2023); therefore, we further observed the effect of quercetin on synovial cells in the rodent ankle joints of model rats. Our in vivo model shows that JAK1/STAT3/HIF-1α signaling inhibits synoviocyte migration and invasion, resulting in a reduction in articular surface destruction and pannus production. It has previously been shown that autoreactive synoviocytes from RA patients need significant quantities of energy and metabolic intermediates during abnormal activation and autoantibody production (Falconer et al. 2018). In RA, the microenvironment is hypoxic, and metabolic shifts occur; HIF-1α acts as a vital transcription factor in RA synoviocytes and is involved in various physiological processes (Fearon et al. 2016), and HIF-1α promotes metabolic reprogramming including anaerobic glycolysis and synoviocyte migration (Koedderitzsch et al. 2021), invasion, apoptosis, and angiogenesis (Liu et al. 2024). Our findings indicate that quercetin can effectively reduce the expression of HIF-1α in synovial fibroblasts at both the mRNA and protein levels. Studies have demonstrated that HIF-1α can activate and induce energy metabolism in synoviocytes; that is, under anaerobic conditions, synoviocytes may select the glycolytic pathway to rapidly obtain energy (Courtnay et al. 2015), HIF-1α performs a vital function in regulating all 12 essential components for glycolysis, including LDHA, PA, and PDH (Zheng et al. 2021). In the normoxic environment, the decomposition of glucose intake takes aerobic respiration as the main metabolic form, and decomposes into pyruvate through the cytoplasmic matrix, acetyl PDH forms CoA and then transferred into mitochondria, participates in the tricarboxylic acid cycle and performs aerobic oxidation for energy as the main way of glucose metabolism decomposition for energy supply, which produces a large amount of ATP to efficiently utilize the glucose ingested by synoviocytes. (Ganapathy-Kanniappan et al. 2013). However, when FLS proliferate in large numbers to form a hypoxic environment, causing aerobic oxidation to shift to activation of glycolysis, FLS requires a large intake of glucose for energy. At the same time, glycolysis can produce large amounts of ATP in a short period compared to aerobic oxidation although the energy supply is low (Garcia-Carbonell et al. 2016), FLS proliferation is similar to “Wabogg effect” proliferation, that is, in the glycolytic environment for the large uptake of glucose energy plunder, and then aggravate synovial hyperplasia, pannus formation and bone invasion. Our study demonstrates that quercetin can reduce glucose uptake, inhibit PA and LDHA production, decrease LA production, and increase PDH production. PDH activity can promote the hydroxylation of HIF-1α and subsequent degradation by the proteome (Selak et al. 2005), thereby inhibiting synovial angiogenesis and leukocyte infiltration, which prevents joint damage. These findings suggest that quercetin regulates glycolysis by downregulating HIF-1α and improving local energy metabolism in RA.

RA-FLS exhibit aggressive characteristics, such as hyperproliferation (Weyand et al. 2017), cell migration (Friščić et al. 2021), invasion (Lee et al. 2023), and apoptotic resistance, all of which require significant energy metabolism (Fearon et al. 2022). Cell proliferation and activation are typically associated with increased glucose metabolism and cytokine secretion, as well as cell migration and proliferation, which decrease when FLS are deprived of glucose or treated with glycolytic inhibitors, reducing arthritis in mice (Garcia-Carbonell et al. 2016). By inducing glycolysis, HIF-1α transcribes genes essential for glucose uptake and glycolysis, thereby regulating energy metabolism to alleviate RA (Courtnay et al. 2015). Our research shows that quercetin significantly impeded the proliferation of MH7A cells by suppressing the action of IL-6 in a concentration-dependent manner. The extensive proliferation of synovial fibroblasts can accelerate their migration and invasion, which can cause tissue and joint injury accompanied by inflammatory infiltration (McGonagle et al. 2017). The data reveal that quercetin significantly inhibited MH7A cell migration and invasion, as well as reduced articular surface damage, which is a result of apoptosis induction (King K L et al. 1998), thereby alleviating the progression of RA disease. One key aspect of RA therapy involves inhibiting the inflammatory response. This is because pro-inflammatory mediators are initially secreted by resident inflammatory cells within the joint, which prompts the marked infiltration of various additional immune cells, causing a damaging feedback cycle involving the release of pro-inflammatory mediators and subsequent RA progression. Our research demonstrates that IL-6 can cause abnormal MH7A cell growth and promote the production of IL-1β, IL-6, IL-17, and TNF-α. Interestingly, quercetin can dose-dependently hinder IL-6-induced MH7A cell proliferation and pro-inflammatory cytokine mRNA expression. These findings indicate that quercetin can regulate various aspects of synoviocyte behavior, which contribute to its potential therapeutic effects in RA; however, the in-depth mechanism remains to be elucidated.

JAK/STAT signaling pathways affect both the morphology and metabolism of synovial fibroblasts (Choi K M et al. 2022). Furthermore, studies in vivo have shown that hypoxia in the synovium can stimulate STAT3 activation and promote synovial fibroblast migration and invasion, with a bidirectional interaction between HIF-1α and STAT3 (Gao et al. 2015). Therefore, our own investigation into the effects of quercetin on JAK1 and STAT3 expression supports these findings, since we observed a decrease in the expression of JAK1, STAT3, and HIF-1α at the protein level in the in vitro model. These data suggest that JAK1, HIF-1α, and STAT3 are closely intertwined and can significantly impact inflammation and joint health. Synovial fibroblasts exhibit a unique active migratory phenotype and strong cartilage invasiveness in RA (M et al. 2019). Both HIF-1α (Hua et al. 2016) and the JAK1-STAT3 (McGarry et al. 2018) signaling pathways play significant roles in the pathogenesis of RA by promoting the migration and invasion of FLS, which leads to cartilage erosion and joint surface destruction. Our in vivo model shows that JAK1/STAT3/HIF-1α signaling inhibits synoviocyte migration and invasion, resulting in a reduction in articular surface destruction and pannus production. These discoveries indicate that inhibiting JAK1/STAT3/HIF-1α signaling can regulate energy metabolism and autoimmune metabolic disorders, leading to decreased pro-inflammatory mediator secretion, reduced synoviocyte migration and invasion, and ultimately slowing of bone and joint destruction. Therefore, it can be hypothesized that quercetin may alleviate RA by regulating energy metabolism via the JAK1/STAT3/HIF-1α signaling axis to inhibit inflammation.

## Conclusion

**Fig. 8:**
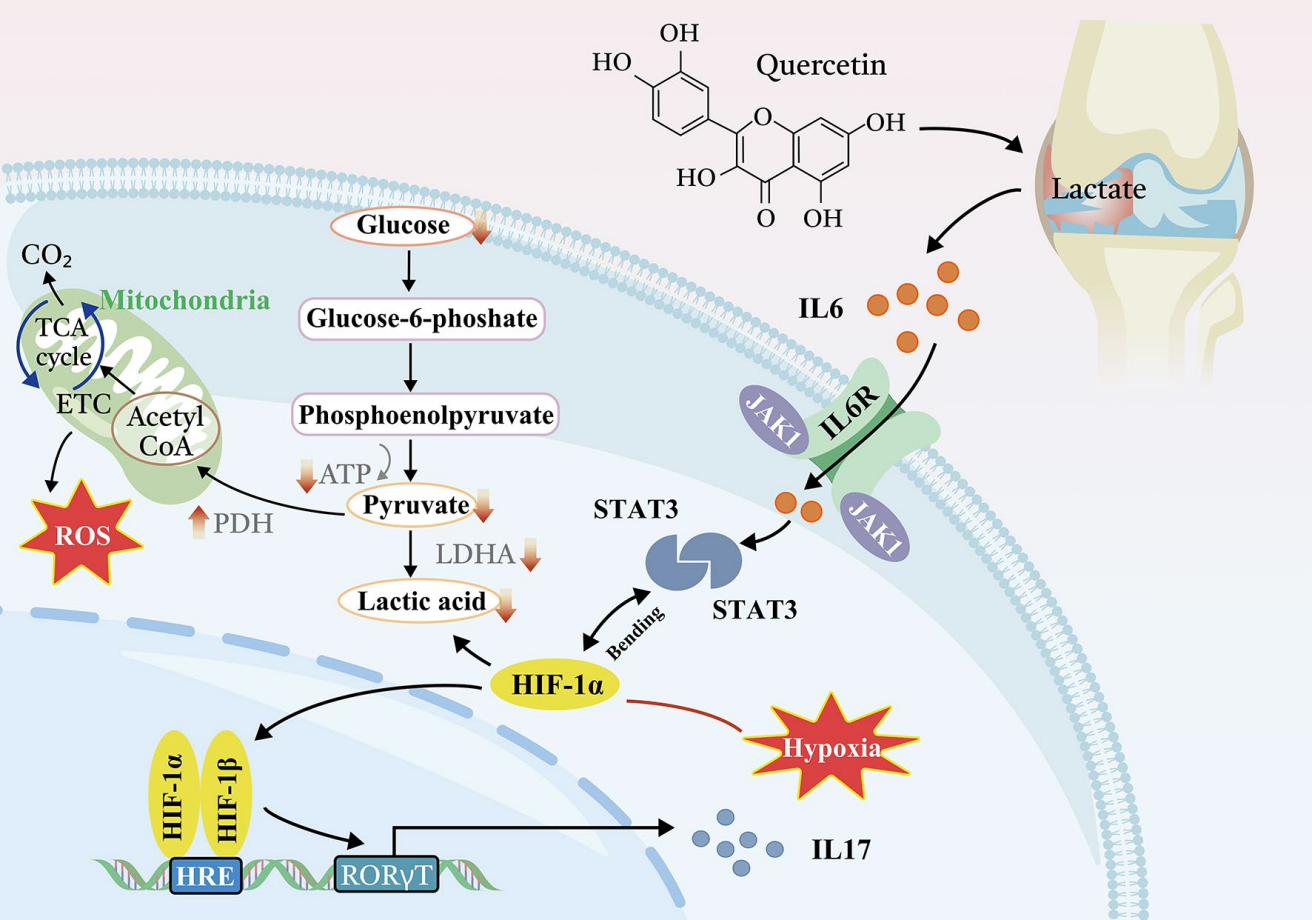
Quercetin’s effect on JAK1/STAT3/HIF-1α signaling in rheumatoid arthritis. The regulatory effect of quercetin on synoviocytes in rheumatoid arthritis mainly involves JAK1/STAT3/HIF-1α signaling and its downstream metabolic and molecular regulation

The mechanism underlying improvements in the condition of CIA rats caused by quercetin is shown in Fig. 8. In this study, quercetin effectively mitigated joint and bone damage as well as arthritis, leading to an overall improvement in the condition of the rats. Our research also highlights the significant role played by HIF-1α in synovial fibroblasts, which helps to address energy metabolism disorders by reducing inflammatory responses through the enhancement of glycolysis. Furthermore, we discovered that JAK1/STAT3 acts upstream of HIF-1α, and quercetin’s impact on JAK1/STAT3/HIF-1α signaling was validated through both in vitro and in vivo testing. Overall, our data suggest that quercetin can potentially treat RA by balancing the immuno-inflammatory system and energy metabolism through modulation of JAK1/STAT3/HIF-1α signaling, providing a new direction in the quest to reveal the positive treatment effects of quercetin in RA.

## Data Availability

No datasets were generated or analysed during the current study.

## Glossary

RA: Rheumatoid arthritis
HT: *Herba taxilli*
Que: Quercetin
JAK1: Janus kinase
STAT3: Signal Transducer and Activator of Transcription 3
HIF-1α: Hypoxia-inducible factor 1α
CIA: Collagen II-induced arthritis
CT: Computed Tomography
CCK8: Cell Counting Kit-8
IL1β: Interleukin 1β
IL6: Interleukin 6
IL17: Interleukin 17
LA: Lactate
LDH: Lactate Dehydrogenase
PDH: Pyruvate Dehydrogenase
PA: Pyruvate
ATP: Adenosine Triphosphate
